# Would placing pictorial health warnings on waterpipe devices reduce waterpipe tobacco smoking? A qualitative exploration of Egyptian waterpipe smokers’ and non-smokers’ responses

**DOI:** 10.1136/tobaccocontrol-2018-054494

**Published:** 2018-07-06

**Authors:** Aya Mostafa, Heba Tallah Mohammed, Wafaa Mohamed Hussein, Mahmoud Elhabiby, Wael Safwat, Sahar Labib, Aisha Aboul Fotouh, Janet Hoek

**Affiliations:** 1 Department of Community, Environmental and Occupational Medicine, Faculty of Medicine, Ain Shams University, Cairo, Egypt; 2 School of Pharmacy, University of Waterloo, Waterloo, Ontario, Canada; 3 Department of Psychiatry Medicine, Faculty of Medicine, Ain Shams University, Cairo, Egypt; 4 Egyptian Tobacco Control Coalition, Cairo, Egypt; 5 Egypt Health Foundation, Cairo, Egypt; 6 Tobacco Control Unit, Ministry of Health, Cairo, Egypt; 7 Departments of Public Health and Marketing, University of Otago, New Zealand

**Keywords:** packaging and labelling, public policy, non-cigarette tobacco products, prevention, cessation

## Abstract

**Background:**

Although Egypt places four generic pictorial health warnings (PHWs) on the front and back half of waterpipe tobacco packs (WTPs), waterpipe tobacco smoking (WTS) rates have continued to rise. It has been suggested that PHWs would be more salient if placed on the waterpipe device itself. This qualitative study explored how participants perceived the effects placing PHWs on waterpipe devices would have on warning salience and uptake or quitting of WTS.

**Methods:**

We conducted 10 focus groups and 10 in-depth interviews with 90 adult waterpipe smokers and non-smokers, men and women, who lived in rural, semi-urban and urban regions of Egypt. We presented participants with four novel PHWs of different sizes positioned randomly at four locations on a waterpipe device (the glass body, metal holder, mouthpiece or hose), one at a time. At each session, participants viewed a PHW on all four locations. Novel warnings were shown on plain labels with a dark uniform background and featured pictures, text and the quitline number. Transcripts were analysed using thematic analysis.

**Results:**

Participants thought placing PHWs on waterpipe devices might increase salience, prevent WTS initiation or trigger quit attempts; they favoured placing PHWs on the glass body, mouthpiece or waterpipe hose. Both waterpipe smoker and non-smoker participants thought these potential effects would affect non-smokers or non-established smokers more than established waterpipe users.

**Conclusions:**

Our exploratory study suggests that PHWs featured prominently on waterpipe devices could potentially deter experimentation with waterpipe tobacco products and promote cessation, especially among non-established users.

## Introduction

Many waterpipe tobacco (WT) users perceive this tobacco to pose less harm than cigarettes,^1^ despite evidence WT is associated with serious diseases.^2^ Flavoured tobacco and lack of regulatory policies have seen waterpipe tobacco smoking (WTS) increase globally,^3^ with WTS prevalence reaching 10% among young adult populations in the USA and UK.^4 5^ Egyptian studies report high WTS prevalence among adolescent girls (3.4%),^6^ university students (12.2%)^7^ and rurally located men (7.5%).^8^ These findings highlight the need for innovative WT control policies to curb its rising use.^9–11^

Tobacco product health warnings can increase awareness of smoking’s risks, foster cessation and deter initiation.^12^ Egypt, a signatory country to the WHO Framework Convention on Tobacco Control (FCTC), has applied a set of four generic pictorial health warnings (PHWs) to the lower front and back half of waterpipe tobacco packs (WTPs) since 2008,^13^ but these still depict colourful fruits and flavours in brand imagery.^14^ Furthermore, WT use differs from cigarette smoking and involves multiple components, including tobacco, charcoal and a device.^11^ A recent WHO report recommended placing PHWs on waterpipe devices, as smokers may not always see WTPs and hence PHWs, particularly at cafés.^9^ Turkey remains the only country to have placed PHWs on waterpipe devices,^9^ though this measure has not been evaluated.

Only two surveys appear to have evaluated placement of PHWs on virtual waterpipe devices.^15 16^ One found that PHWs had a modest impact on established waterpipe smokers in the USA, with warning locations on the base, mouthpiece and stem of the watrpipe device having similar visibility.^15^ Another reported that existing WTP PHWs in Egypt lacked visibility, while placing warnings on the waterpipe device itself, particularly the mouthpiece, increased visibility.^16^

These findings suggest that Governments should respond more directly to the FCTC and the WHO’s recent report, especially in countries where WTS and device manufacturing are commonplace.^9^ To guide policy development, we used a qualitative approach to explore how participants perceived the effects placing PHWs on waterpipe devices would have on warning salience and uptake or quitting of WTS.

## Methods

### Design

We conducted 10 focus groups and 10 in-depth interviews between 2015 and 2016 at the Faculty of Medicine, Ain Shams University, and at participants’ homes or in cafés.

### Sample

Our sample comprised men and women aged ≥18 years, self-identified waterpipe smokers (exclusive WT or dual users of WT and cigarettes) and non-smokers (non-users of any tobacco product), who lived in urban, semi-urban Cairo and rural Menoufia. As WT use in Egypt is generally higher among men,^8^ more men participated in our sessions. In total, 90 participants were recruited using snowball sampling^17^; 80 participated in focus groups (structured with respect to age, gender, smoking status and comprising 6–8 individuals per group) and 10 participated in in-depth interviews (see the online supplementary tables 1 and 2).

10.1136/tobaccocontrol-2018-054494.supp1 Supplementary file 1

### Tools

We developed the interview guide^18^ in Egyptian colloquial Arabic and pilot tested it for clarity and comprehensiveness. We used the same guide during focus groups and in-depth interviews to probe participants’ experiences of WTS, their knowledge of WT PHWs, their views on the existing and novel PHWs on WTPs, and their perceptions of the effects PHW placement on waterpipe devices would have. In this article, we address the latter topic.

**Figure 1:**
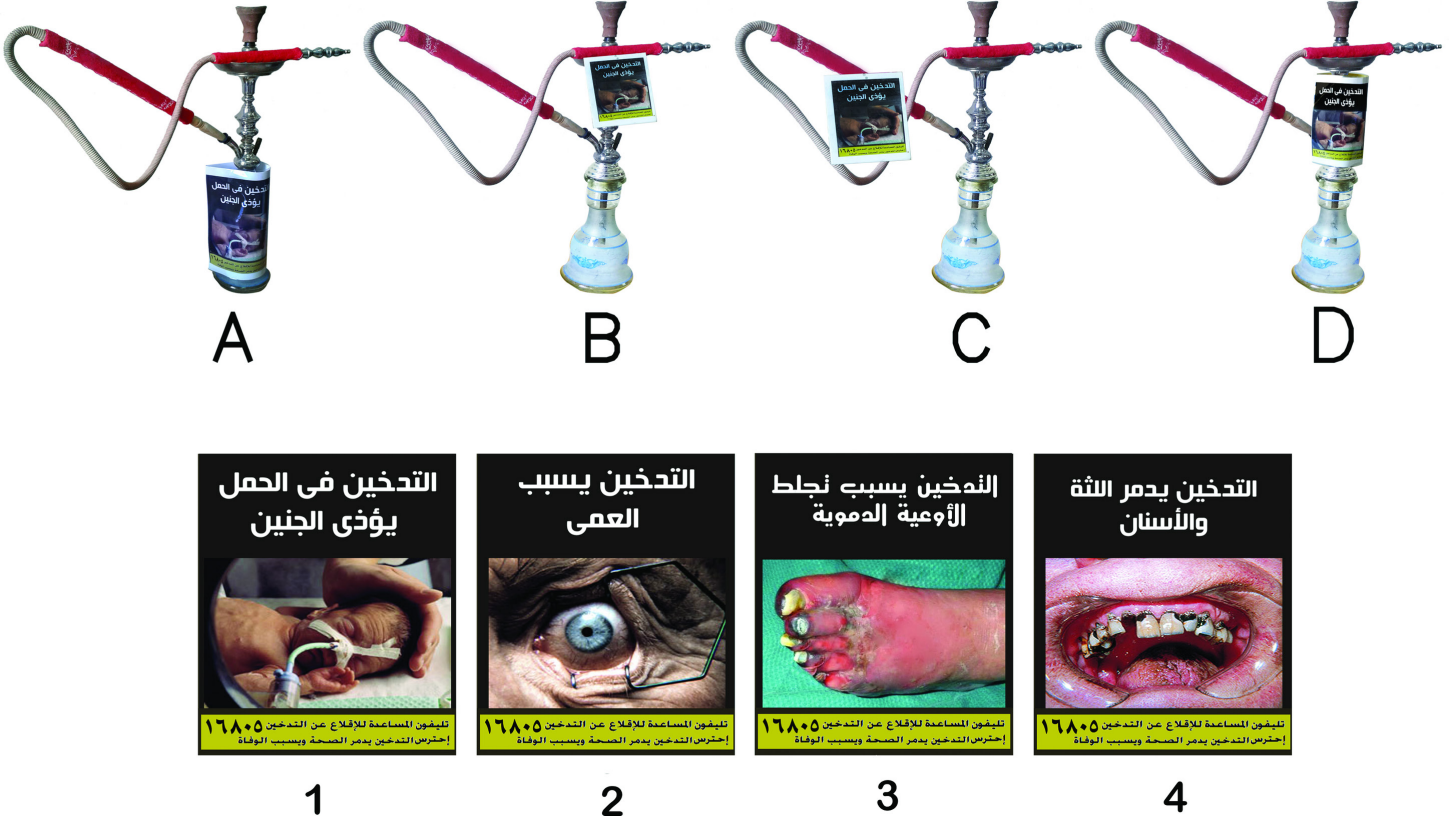
Example of novel PHWs placed on four spots of the waterpipe device: (A) glass body, (B) mouthpiece, (C) hose, (D) metal holder. Novel PHWs used in this study: (1) fetal harm, (2) blindness, (3) blood vessel clotting, (4) teeth and gum decay. PHW, pictorial health warning.

We adapted four novel PHWs from a health warning database^19^ and drew on the Tobacco Control Unit’s (Egyptian Ministry of Health) suggestion to introduce large PHWs and use a plain format as recommended by the WHO FCTC.^20 21^ Novel warnings included pictures, text and the quitline number and were designed against a dark uniform plain background. Each of the four novel PHWs were prepared in four different sizes to match the suggested locations on an actual waterpipe device (the glass body, metal holder, mouthpiece or hose) (figure 1) and enveloped in transparent flexible hard plastic wrap to maintain quality throughout the sessions.

### Data collection

All participants provided verbal consent prior to each session and were assured their data and identity would remain confidential. Each audiorecorded focus group or interview was moderated by two of the co-authors (AM, WS, ME and WMH) and lasted about an hour.

Participants viewed four novel PHWs placed at random on four different locations of a waterpipe device: the glass body, metal holder, mouthpiece or hose, one at a time. After viewing a PHW on one location, participants commented on PHW salience and potential effect on WTS uptake or cessation before they were presented with a PHW on another location of the device. At each session, we presented PHWs and locations at a random order; all participants viewed a PHW on all four locations. The facilitator and note taker regularly switched roles to promote reflection, and the wider team critically reviewed the interviews. No further sessions were scheduled once thematic saturation had been reached.^22^

### Analysis

Two authors independently transcribed verbatim the recorded sessions and compared the two transcripts to ensure inclusivity and accuracy (WMH, HM); a third author (AM) resolved any discrepancies. Data from focus groups and interviews were aggregated and analysed together using a three-phase thematic approach^23 24^:organising ideas in relation to the research questions, identifying preliminary themes and creating an initial coding list.^25^ We refined this list (AM, AA), added new codes where appropriate and developed broader themes; one author (HM) then reviewed these. We resolved minor inconsistencies before finalising the themes. We present below combined findings from both focus groups and interviews and cite exemplar quotations (see also the online supplementary table 3).

## Results

### Most salient location of PHWs on waterpipe devices

Participants showed similar preference for each location tested, though the metal holder was the least favoured location. Some participants queried the ease with which warnings could be placed on parts of the waterpipe device, given variations in device shape and size. Several suggested the glass base could be a logical site because it is large, fixed and often closely monitored by smokers for water level and colour, and when cleaning the device. However, others thought this location was impractical and would impede smokers’ ability to check the water clarity and level and, in some cases, may be hidden under the table or placed behind the smoker, out of sight. Furthermore, warnings could be damaged or detached when the glass body is washed.

Those who favoured mouthpiece or hose locations noted that warnings placed on these sites would be visible to smokers as well as to others: ‘*The mouthpiece is the nearest visible part to me…also other people sitting around me will see it in my hand*’ *(male smoker, >25 years, semi-urban).* Smokers holding the mouthpiece in their hands, or dangling the hose beside them, would be exposed repeatedly to the PHW throughout a smoking session: ‘*The smoker will find it just in their face! It will be in their eyesight all the time*’ *(male smoker, >25 years, semi-urban)*. Some smokers reported that they would be disgusted to use mouthpiece with a warning attached to it: ‘*I won’t put this in my mouth!*’ *(female smoker, <25 years, urban)*. However, others noted that the mouthpieces and hoses were disposable, and so could be replaced if users wanted to avoid warnings. Some suggested placing warnings on the metal stem, which was near the charcoal carrier; however, others thought the burning charcoal could damage warnings placed there.

### Perceived likely effects of placing PHWs on waterpipe devices on uptake or quitting of WTS

In general, waterpipe smoker and non-smoker participants thought placing warnings on waterpipe devices would increase awareness of WTS hazards, promote quitting: ‘*If warnings were placed on the device, I may quit smoking’ (male smoker, >25 years, urban)* and deter non-smokers: *“That will attract my attention…It will be more effective among non-smokers who will find it a bit strange and will start talking about it with the smokers they know everywhere…at home…in cafes*’ *(male non-smoker, <25 years, rural)*. Some smokers felt that placing warnings on the device might help them quit or forgo a smoking session: *‘No one will desire to smoke (if warnings were put on the device)…they’ll tell you: get this shisha away from my face…and smoking shisha will decrease*’ *(male smoker, >25 years, semi-urban)*.

However, participants thought these potential effects might be more likely in non-smokers or non-established waterpipe users rather than regular waterpipe smokers. Waterpipe smokers shared this view: ‘It *could affect those who want to start smoking, but the older smokers won’t be affected much*’ *(male smoker, <25 years, semi-urban);* ‘*Pictures draw attention, but I didn’t care because I don’t have a will to quit*’ *(female smoker, >25 years, semi-urban)* as did non-smokers: ‘*It will be more effective among non-smokers rather than among smokers who usually don’t care*’ *(male non-smoker, <25 years, rural);* ‘*Warnings on shisha will have some effect, it may not make smokers quit, but it may make them reduce smoking*’ *(male, non-smoker, <25 years, rural).*

## Discussion

Participants thought that placing PHWs on waterpipe devices would increase salience, quitting or reduction, and prevention of WTS initiation, and believed these effects would be greater among non-smokers or non-established waterpipe users. They considered established waterpipe smokers would be less likely to change their WT use, though thought some may forgo smoking or reduce their WTS consumption. These findings substantiate earlier PHW research with waterpipe^15^ and cigarette^26^ users.

An average WTS session is 30–45 min; if prominently placed, PHWs could ensure that both waterpipe smokers and others nearby have repeated exposure to warnings. Our findings concur with previous research that placing PHWs on the glass body, hose or mouthpiece would be among the most noticeable locations.^15 16^

Policy-makers should consider enacting legislation to require PHWs on waterpipe devices, as these will be seen by both café and home users, and across urban and rural settings. Such a policy might be particularly effective in rural areas of Egypt, where WTS rates are higher than urban areas.^8^ Rural residents mainly smoke WT at home, therefore, would be more frequently exposed to the device during both preparation and commencing the WTS session. Legislation should recognise the various sizes and shapes that waterpipe devices have and consider Turkey’s experience (where PHWs are required to cover 65% of both sides of the glass bowl surface, with stipulated fines for violations).^9^ To strengthen implementation, cafés serving waterpipes could be required to place PHWs on their waterpipe devices as a condition for obtaining and renewing licences.

We explored projected rather than real-life responses to PHWs and used generic rather than waterpipe-specific warning labels, indicating a need for further evaluation of this policy measure. Our sample limits the generalisability of our findings, though a sample of 90 individuals is substantial for qualitative research and saturation was reached in the responses received. Despite these limitations, our findings provide novel insights into the potential salience and perceived effects of placing PHWs on waterpipe devices, extend earlier work as we collected data from waterpipe smokers and non-smokers in a country where WTS is prevalent, even though WT PHWs have been in place for a decade, and reinforce calls for a comprehensive WT regulatory framework.

**What this paper adds**

**What is already known on this subject**

- Pictorial health warnings (PHWs) have the potential to raise awareness of hazards from waterpipe tobacco smoking.
- The WHO’s Framework Convention on Tobacco Control recommendations with respect to waterpipe tobacco labelling regulations are poorly implemented.

**What important gaps in knowledge exist on this topic**

- Few studies have examined how featuring PHWs on waterpipe devices would affect smokers or non-smokers, or the locations most likely to have visual impact.

**What this study adds**

- Waterpipe tobacco labelling regulations should require waterpipe devices to feature PHWs in visually prominent positions.

## References

[1] Jawad M, McEwen A, McNeill A, et al To what extent should waterpipe tobacco smoking become a public health priority? Addiction 2013;108:1873–84. 10.1111/add.12265 23863044

[2] Waziry R, Jawad M, Ballout RA, et al The effects of waterpipe tobacco smoking on health outcomes: an updated systematic review and meta-analysis. Int J Epidemiol 2017;46:32–43. 10.1093/ije/dyw021 27075769

[3] Maziak W, Taleb ZB, Bahelah R, et al The global epidemiology of waterpipe smoking. Tob Control 2015;24 (Suppl 1):i3–i12. 10.1136/tobaccocontrol-2014-051903 PMC4345835 25298368

[4] Cobb C, Ward KD, Maziak W, et al Waterpipe tobacco smoking: an emerging health crisis in the United States. Am J Health Behav 2010;34:275–85. 10.5993/AJHB.34.3.3 20001185 PMC3215592

[5] Grant A, Morrison R, Dockrell MJ Prevalence of waterpipe (Shisha, Narghille, Hookah) use among adults in Great Britain and factors associated with waterpipe use: data from cross-sectional Online Surveys in 2012 and 2013. Nicotine Tob Res 2014;16:931–8. 10.1093/ntr/ntu015 24550183

[6] El Awa F, Fouad H, El Naga RA, et al Prevalence of tobacco use among adult and adolescent females in Egypt. East Mediterr Health J 2013;19:749–54. 10.26719/2013.19.8.749 24975361

[7] World Health Organization. Shisha and smokeless tobacco use among university students in Egypt: prevalence, determinants, and economic aspect: A joint report by the Egyptian Ministry of Health and Population and the World Health Organization, 2014 http://applications.emro.who.int/dsaf/EMROPUB_2014_EN_1752.pdf?ua=1&ua=1 (accessed May 2017).

[8] World Health Organization. Global Adult Tobacco Survey: Egypt Country Report. 2009 http://www.who.int/tobacco/surveillance/gats_rep_egypt.pdf (accessed Sep 2017).

[9] World Health Organization. Framework Convention on Tobacco Control: Control and prevention of waterpipe tobacco products (document FCTC/COP/7/10). In: Conference of the parties to the WHO framework convention on tobacco control, seventh session, Delhi, India, 7-12 November 2016: World Health Organization, Geneva http://www.who.int/fctc/cop/cop7/FCTC_COP_7_10_EN.pdf (accessed May 2017).

[10] Jawad M, El Kadi L, Mugharbil S, et al Waterpipe tobacco smoking legislation and policy enactment: a global analysis. Tob Control 2015;24 (Suppl 1):i60–i65. 10.1136/tobaccocontrol-2014-051911 PMC4345984 25550418

[11] Salloum RG, Asfar T, Maziak W Toward a Regulatory Framework for the Waterpipe. Am J Public Health 2016;106:1773–7. 10.2105/AJPH.2016.303322 27552262 PMC5024375

[12] Hammond D Health warning messages on tobacco products: a review. Tob Control 2011;20:327–37. 10.1136/tc.2010.037630 21606180

[13] Mostafa A, Kashiwabara M Tobacco packaging and labelling policies in countries of the Eastern Mediterranean and Western Pacific Regions: Post-deadline assessment of the time-bound measures of WHO FCTC Article 11. Tob Prev Cessat 2016;2:78 10.18332/tpc/66793

[14] World Health Organization. Tobacco control country profiles. 2017 Egypt http://www.who.int/tobacco/surveillance/policy/country_profile/egy.pdf?ua=1 (accessed 15 Dec 2017).

[15] Islam F, Salloum RG, Nakkash R, et al Effectiveness of health warnings for waterpipe tobacco smoking among college students. Int J Public Health 2016;61:709–15. 10.1007/s00038-016-0805-0 26971508 PMC4992403

[16] Mostafa A, Mohammed HT Graphic health warnings and their best position on waterpipes: A cross-sectional survey of expert and public opinion. Tob Prev Cessat 2017;3:116 10.18332/tpc/70873 PMC7232823 32432191

[17] Feldman MS, Bell J, Berger MT Gaining Access: A Practical and Theoretical Guide for Qualitative Researchers. Oxford, UK: Altamira Press, 2003.

[18] IARC Handbooks of Cancer Prevention, Tobacco Control. Methods for Evaluating Tobacco Control Policies. Lyon, France, 2008:12 http://www.iarc.fr/en/publications/pdfs-online/prev/handbook12/Tobacco_vol12.pdf (accessed Apr 2015).

[19] World Health Organization Framework Convention on Tobacco Control. Health Warnings Database. http://www.who.int/tobacco/healthwarningsdatabase/en/ (accessed Apr 2015).

[20] World Health Organization. Guidelines for implementation of Article 11 of the WHO Framework Convention on Tobacco Control (Packaging and labelling of tobacco products). 2008 http://www.who.int/fctc/guidelines/article_11.pdf?ua=1 (accessed Apr 2015).

[21] World Health Organization. Evidence, Design and Implementation of Plain Packaging at. http://apps.who.int/iris/bitstream/10665/207478/1/9789241565226_eng.pdf?ua=1 (accessed Dec 2016).

[22] Walker JL Research column. The use of saturation in qualitative research. Can J Cardiovasc Nurs 2012;22:37–46. 22803288

[23] Braun V, Clarke V Using thematic analysis in psychology. Qual Res Psychol 2006;3:77–101. 10.1191/1478088706qp063oa

[24] Pope C, Ziebland S, Mays N Qualitative research in health care. Analysing qualitative data. BMJ 2000;320:114–6. 10.1136/bmj.320.7227.114 10625273 PMC1117368

[25] Boyatzis RE Transforming qualitative information: thematic analysis and code development. California: Sage Publications, 1998.

[26] Munafò MR, Roberts N, Bauld L, et al Plain packaging increases visual attention to health warnings on cigarette packs in non-smokers and weekly smokers but not daily smokers. Addiction 2011;106:1505–10. 10.1111/j.1360-0443.2011.03430.x 21401767

