# Supplementary material for: Would placing pictorial health warnings on waterpipe devices reduce waterpipe tobacco smoking? A qualitative exploration of Egyptian waterpipe smokers’ and non-smokers’ responses
Source: Tob Control. 2018 Jul 6;28(4):475–8. doi: 10.1136/tobaccocontrol-2018-054494 (PMC6589487; doi:10.1136/tobaccocontrol-2018-054494)
Supplement: Supplementary file 1 [file tobaccocontrol-2018-054494supp001.pdf]

Supplementary File

Table 1. Total number of interviews and focus group discussions conducted by demographic profile of participants

	FGDs (10, total participants=80)				IDIs (10)			
	Males (7)		Females (3)		Males (7)		Females (3)	
Age	S	NS	S	NS	S	NS	S	NS
	(5)	(2)	(2)	(1)	(6)	(1)	(2)	(1)
18-24	2	1	1		3	1	1	
25+	3	1	1	1	3		1	1

FGDs=Focus group discussions, IDIs=In-depth interviews, S=Waterpipe smoker, NS=Non-smoker

Table 2. Sociodemographic characteristics and waterpipe smoking status of participants

	N=90	%
**Gender**		
Male	**65**	**72.2**
Female	**25**	**27.8**
**Age (range 18-73)**		
**Mean age ± standard deviation** 33.4 ± 11.6		
**Age group, years**		
18-24	**21**	**23.3**
25+	**69**	**76.7**
**Education level**		
Primary or lower	**16**	**17.7**
Secondary	**32**	**35.6**
Tertiary	**42**	**46.7**
**Occupation**		
Employed professional	**42**	**46.7**
Employed non-professional	**34**	**37.8**
In further study or seeking employment	**14**	**15.5**
**Residence**		
Rural	**20**	**22.2**
Semi-urban	**51**	**56.7**
Urban	**19**	**21.2**
**Smoking status**		
Waterpipe smoker	**62**	**68.9**
Non-smoker	**28**	**31.1**
**Ever noticed PHWs on WTPs**		
Yes	**53**	**58.9**
**Where**		
At cafés	**17**	**32.1**
At home	**11**	**20.8**
Buying tobacco	**25**	**47.2**

Table 3. Selected participants' quotes representing views of placement of warnings on the waterpipe device. Quotes are presented by gender, age group*, smoking status, and region of participants

"If the warnings were printed on the glass part, I would never buy it"	*(Male smoker, >25y, rural)*
"Most of us have a shisha at home…this glass part…we wash it frequently…and this will make one see it (the warning) all the time"	*(Male smoker >25y, rural)*
"The glass part is the best place, the bigger the warning the better"	*(Female smoker, >25y, semi-urban)*
"The water container is the best place, most smokers check it frequently"	*(Male smoker, <25y, semi-urban)*
"A warning should be put on the glass part, it would be big enough…it may be not quite visible under tables though"	*(Female non-smoker, >25y, semi-urban)*
"It will be difficult to put warnings on the glass part as smokers tend to observe the water level inside to see if needs to be changed"	*(Male smoker, >25y, semi-urban)*
"Warnings can be printed on the glass part, while allowing for observing water level…so that if the water container is washed the warnings won't be affected"	*(Male smoker, >25y, semi-urban)*
"The water container is the best, however if the warning is removable, smokers would remove it to be able to check the water, so the warning must be well attached to the container or even printed"	*(Male smoker, <25y, semi-urban)*
"There are some expensive forms of devices presented in cafes of various shapes and have colored water, it would be difficult to put warnings there"	*(Male non-smoker, >25y, semi-urban)*
"The warning on the glass part will be at the feet of a smoker, he won't see it"	*(Male non-smoker, <25y, rural)*
"The mouth piece is a good place, smokers may feel disgusted to put it in their mouth"	*(Male smoker, >25y, semi-urban)*
"I think the loop is best place because it is closer to a smoker when it is being held in his hand"	*(Male smoker, >25y, semi-urban)*
"The mouthpiece and hose are available now as one piece that is disposable"	*(Male smoker, <25y, urban)*
"Now, there are what they call 'medical loops' (referring to disposable plastic loops), that can be easily changed"	*(Male non-smoker, >25y, semi-urban)*
"The loop can be disposable now, so how will warnings be attached to it"	*(Male smoker, >25y, semi-urban)*
"It can be printed on menus … "	*(Male non-smoker, >25y, semi-urban)*
"Warnings on shisha devices will increase awareness"	*(Male, non-smoker, <25y, rural)*
"If warnings are placed on the device, I may quit smoking"	*(Male smoker, >25y, urban)*
"It will be more effective among nonsmokers rather than among smokers who usually don't care"	*(Male non-smoker, <25y, rural)*
"Warnings on waterpipe device will have some effect, it may not make smokers quit but it may make them reduce"	*(Male non-smoker, <25y, rural)*

“This would attract the attention of those who don’t smoke shisha, it would be something odd for them to see”	*(Female smoker, <25y, urban)*
"If I were a smoker and have seen this picture, I won't quit but at least wouldn't smoke at the moment, the picture is disgusting”	*(Male non-smoker, <25y, rural)*
“Warnings influence the non-smokers much more than smokers, as non-smokers usually find the picture more disgusting”	*(Female, non-smoker, >25, semi-urban)*
"A beginner may be affected by the warnings…”	*(Female smoker >25 Semi-urban)*
"If someone wants to smoke, they will smoke anyway and the pictures make no difference”	*(Male, smoker, <25y, semi-urban)*
"I have seen the warnings too often that they no longer frighten me”	*(Male smoker, <25y, rural)*
"If I saw the warnings on the device I may refuse to smoke and ask for it to be removed out of my sight”	*(Male, smoker, >25y, semi-urban)*
"It will take people 2 to 3 days and they will get used to the new warnings”	*(Male, smoker, >25y, urban)*

*The age group of participants was categorized as > or < 25 years, because we were interested in highlighting the views of young adults who may be at higher risk of experiencing WTS or maybe non-established WT users yet, versus older adults who might more likely have established a smoking or non-smoking behaviour.
